# The Relationship between Poor Pulmonary Function and Irregular Pulse in the Elderly: Findings from a Nationwide Cross-Sectional Survey in Korea

**DOI:** 10.3390/healthcare8030312

**Published:** 2020-09-01

**Authors:** Sun Hwa Kim, Yonggu Lee, Seon Young Hwang, Jinho Shin, Chun Ki Kim, Jin-Kyu Park

**Affiliations:** 1 Department of Nursing, Hanyang University Medical Center, Seoul 04763, Korea; 79ssunhwa@gmail.com; 2 Division of Cardiology, Hanyang University Guri Hospital, Guri 11900, Korea; hmedi97@naver.com; 3 College of Nursing, Hanyang University, Seoul 04763, Korea; seon9772@hanyang.ac.kr; 4 Division of Cardiology, Department of Internal Medicine, College of Medicine, Hanyang University Medical Center, Seoul 04763, Korea; jhs2003@hanyang.ac.kr; 5 Department of Medicine, Hanyang University College of Medicine, Seoul 04763, Korea; chunkikim@hanyang.ac.kr

**Keywords:** arrhythmia, atrial fibrillation, physical examination, pulmonary function test, pulse rate

## Abstract

Arrhythmia may be caused by reduced pulmonary function, and pulse palpation is a useful screening method for the early detection of cardiac arrhythmia. The aim of this study was to investigate the association between reduced pulmonary function and abnormal findings on pulse palpation in 2347 subjects aged ≥65 years using data from a nationwide survey. Pulse palpation was initially performed for 15 s and, if felt to be abnormal, it was performed again for 60 s. The prevalence of irregular pulse (IP) determined by the 60-second palpation was 61 (2.6%). The mean age of subjects with an IP was 73.0 (95% CI 71.7–74.3) years, and 45.8% were male. After adjustment for covariates, forced vital capacity (FVC)/predicted FVC, forced expiratory volume in one second (FEV_1_)/predicted FEV_1_, and the lowest FEV_1_ remained significant risk factors for IP. A restrictive or obstructive spirometry pattern was also an independent risk factor for IP. In summary, an IP is more prevalent when pulmonary function is reduced in the elderly, in whom careful pulse palpation may be necessary for the early detection of arrhythmia.

## 1. Introduction

Some cardiac arrhythmias do not cause any symptoms and may first be discovered only incidentally during a routine examination. Some patients are unaware of the presence of arrhythmia, even when it is chronic. Therefore, early detection and treatment of arrhythmias are critical for minimizing associated complications. One of the most common arrhythmias in the elderly is atrial fibrillation (AF). This condition increases the risk of stroke five-fold and the risk of cardiac failure three-fold, leading to an increase in the mortality rate [1,2,3]. Patients usually recognize the onset of paroxysmal AF due to feeling palpitations with a sensation of a rapid heartbeat [4]. An irregular pulse (IP) can also be a sign of an arrhythmia [5].

An IP on physical examination is not a rare finding in primary care settings. Pulse palpation is a useful screening method for the early detection of cardiac arrhythmias [5,6] and is routinely included in physical examinations by a clinical nurse. Opportunistic screening for early AF detection by pulse palpation in subjects 65 years of age or older is recommended by the European Society of Cardiology guidelines [7]. This recommendation highlights the importance of appropriate and timely examination of the elderly for the early detection and prevention of stroke and heart disease. Systematic population screening is useful for identifying individuals with AF, but the use of electrocardiograms (ECGs) to screen for AF may be expensive and time-consuming [8]. The Screening for Atrial Fibrillation in the Elderly (SAFE) study concluded that opportunistic screening, such as pulse palpation and optional ECG, is the most appropriate screening method for those with IP [9]. Firzmaurice et al. also reported that pulse palpation is a cost-effective approach [10].

The heart and lungs have highly related hemodynamics; patients suffering from chronic obstructive pulmonary disease (COPD) are often found to have arrhythmias (including AF). In cases of COPD with insufficient pulmonary function, deterioration of gas composition, and pulmonary hypertension may result in elevated atrial pressure and altered electrophysiological properties of atrial tissue and can trigger ectopic beats and supraventricular tachycardia [11,12]. In addition, it has previously been reported that a low forced expiratory volume in one second (FEV_1_) is related to an increased risk of AF [13]. The risk of AF was 1.8 times higher for those with an FEV_1_ between 60% and 80%, and reduced lung function has been reported to be an independent predictor of AF [14]. Therefore, subjects with reduced lung function may benefit from more judicious screening for cardiac arrhythmia. 

While pulse palpation is a useful screening method for the early detection of cardiac arrhythmias and has a high sensitivity for atrial fibrillation [5,6,10], the utility of pulse palpation or the prevalence of IP in subjects with reduced pulmonary function relative to those with normal pulmonary function is not yet known. Therefore, we aimed to examine the relationship between pulmonary function and irregular pulse (IP) in people aged 65 or older.

## 2. Materials and Methods

### 2.1. Study Design

A cross-sectional descriptive survey design using a secondary analysis of national data was adopted for this study to identify the relationship between reduced pulmonary function and IP.

### 2.2. Setting and Sample

The present study was performed using nationally representative data from the 5^th^ Korea National Health and Nutrition Examination Survey (KNHANES V), a government-approved statistical survey performed between 2010 and 2012 by the Korean Centers for Disease Control and Prevention [15]. The survey was released and made available for download from the KNHANES website (https://knhanes.cdc.go.kr/) after its approval for use. A rolling sampling design for the survey was employed, which involved a stratified, complex, multi-stage, probability-clustered survey on a sample representing the community-dwelling civilian population in South Korea. The survey was distributed to 11,400 households in 568 national districts. The enumeration districts were created by separating the country into 11 regions. These regions were subdivided into 26 regional layers based on population composition by age. The survey comprised three components: (1) a nutrition survey for dietary assessment, (2) a health examination survey on common cardiovascular diseases and chronic diseases such as cancer, pulmonary disease, arthritis, and so on, and (3) a health interview survey on general health conditions and health-related lifestyle.

Of all subjects aged ≥65 years in the KNHANES database, 2347 subjects who completed the health examination survey and had spirometry data were included in this study.

### 2.3. Measurements

#### 2.3.1. General Characteristics

A self-administered questionnaire was used to evaluate the history of cigarette smoking (current smoker, ex-smoker, or non-smoker), consumption of alcohol, and level of physical activity (walking, moderate, or strenuous activity), and the data were collected by interviewers. Those who smoked fewer than 100 cigarettes in their lifetime were considered non-smokers, while those who smoked more than 100 cigarettes were classified as ex- or current smokers. The frequency of alcohol consumption was divided into four categories based on the average number of drinks in the one-year period before the survey (on average 1 or less per month, 2–4 times per month, 2–3 times per week, or 4 or more times per week). The average amount of alcohol intake in a single session was also classified into four categories, i.e., none, 1–4 servings, 5–9 servings, or ≥10 servings per session.

Physical activity was classified into 3 categories: walking regularly for 30 min or longer in indoor or outdoor at least 5 times a week was categorized as ‘Walking’, exercising for 30 min or longer by carrying a light item at least 3 times a week was categorized as ‘Moderate activity’, and activities such as climbing, running, fast biking, and carrying heavy objects for 20 min or longer at least 3 times per week were categorized as ‘Strenuous activity’ means.

#### 2.3.2. Physiological Data

Body weight, height, and waist circumference were measured by well-trained examiners. Body weight was recorded to the nearest 0.1 kg using a calibrated balance-beam scale (Giant-150N; Hana Co. Ltd., Seoul, Korea). A portable stadiometer (850–2060 mm; Seriter, Bismarck, ND, USA) was used to measure height to the nearest 1 mm. Waist circumference was measured and recorded to the nearest 0.1 cm in a horizontal plane at the midpoint between the iliac crest and the costal margin at the end of a normal expiration. Body mass index (BMI) was estimated as body weight (kg) divided by height squared (m^2^).

Blood samples were taken using a venipuncture during the health examination after an overnight fast of at least 8 h. Total cholesterol, serum glucose, triglycerides, creatinine, and high-density lipoprotein (HDL) cholesterol were enzymatically assessed. Blood pressure and pulse rate were measured by a nurse on the professional checkup team within the Korean Centers for Disease Control and Prevention. Blood pressure (BP) was measured three times using mercury sphygmomanometers (Baumanometer; Baum, Copiague, NY, USA) while subjects sat quietly after a 5-minute rest. The final BP value was calculated by taking the average value of the second and third BP measurements.

#### 2.3.3. Pulse Palpation

The radial pulse was palpated initially for 15 s after the subjects had rested for five minutes in a seated position. If the pulse was felt to be not completely normal, e.g., irregular, slow (less than 15 beats), or fast (more than 26 beats), the pulse palpation was performed again for 60 s. Subjects were finally considered to have an “IP” if there were variations in the rhythm of pulses in the radial artery during 60-second palpation.

#### 2.3.4. Spirometry Measurement

Spirometry was performed using a dry rolling-seal spirometry device (Vmax series Sensor Medics 2130; Sensor Medics, Anaheim, CA, USA) that was operated by specially trained technicians who complied with the pulmonary function test (PFT) guidelines by the American Thoracic Society and European Respiratory Society [16]. Participants with FEV_1_/forced vital capacity (FVC) of <0.7 were considered to have COPD. The study only used spirometry results that contained two or more acceptable curves that met the reproducibility criteria [16].

### 2.4. Data Analysis

Statistical analyses were carried out using the Statistical Package for the Social Sciences (SPSS, IBM, Armonk, NY, USA) program, version 18.0. The sample weights were incorporated, and the analyses were adjusted for the survey’s complex sample design. We weighted the survey samples in all of the analyses to generate the estimations that represented the Korean non-institutionalized civilian population. More details regarding how the adjustment was performed are available in a previous study [15]. Continuous variables are represented by mean values with confidence intervals (CIs) of 95%, and categorical variables are represented by frequencies and percentages. The chi-square test was employed to analyze the categorical variables. The differences in the mean values were evaluated by Student’s t-tests. A multivariate binary logistic regression analysis was performed to assess the independent relationship between the spirometry results or the history of COPD and IP. Each of 6 parameters of spirometry including FVC (categorical), FVC/predicted FVC (pFVC) (continuous), FVC/pFVC ≥80% (binary), FEV_1_ (categorical), FEV_1_/predicted FEV_1_ (pFEV_1_) (continuous), and the interpretation of the spirometry results (categorical; normal versus restrictive versus obstructive) were evaluated in the multivariate models. We first assessed the strength of associations between IP and potential confounding variables, including age, sex, BMI, waist circumference, alcohol consumption, chronic diseases (hypertension, diabetes, congestive heart failure, angina, previous myocardial infarction, hypercholesterolemia, hypertriglyceridemia and hypo-HDL cholesterolemia), smoking status, thyroid disease, medications (antihypertensive, antidiabetic, and lipid-lowering drugs), and physical activity, using univariate binary logistic models. Of these variables, those with *p* < 0.1 were selected and included in the multivariate binary logistic models as covariates. Then, multivariate binary logistic models for the presence of IP were generated with each parameter of the spirometry results or the history of COPD and the selected covariates. FVC and FEV_1_ were evenly divided into 4 levels based on their respective quartile values, and the adjusted odds ratio (aOR) and 95% CI for each level of the variables were estimated against their respective highest quartile level as the reference. A two-tailed *p*-value of less than 0.05 was considered statistically significant.

## 3. Results

### 3.1. General Characteristics

**Figure 1:**
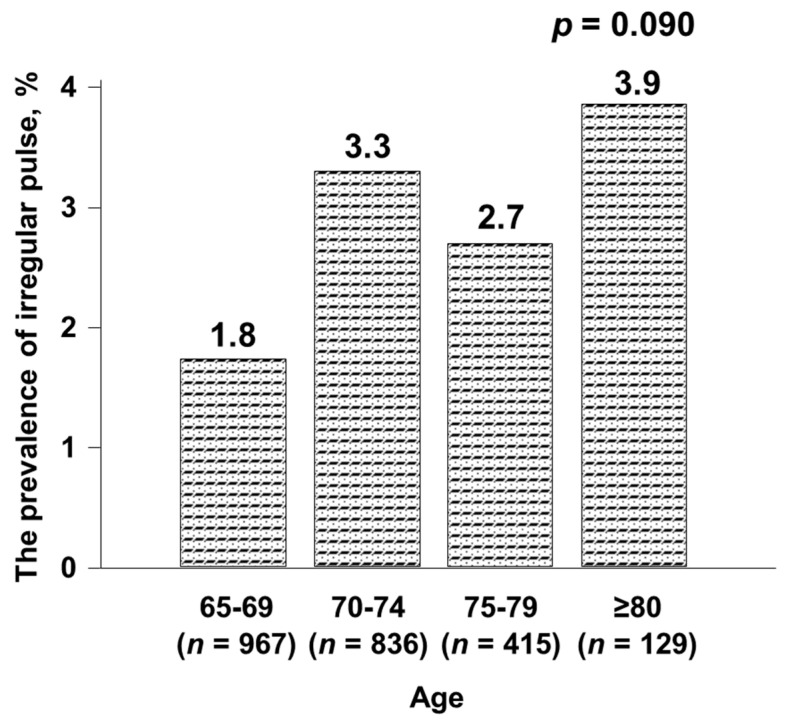
Prevalence of irregular pulse in the general population ≥65 years old.

The general characteristics of all subjects are shown in Table 1. Among the 2347 subjects, 61 (2.6%) were judged to have an IP (hereafter referred to as the IP group). Figure 1 shows the prevalence of IP stratified by age. The highest frequency of IP (3.9%) was seen in those older than 80 years of age, while the prevalence of IP tended to increase with age (*p* = 0.090). Smoking status was not significantly different between the two groups. However, there was a significant difference in alcohol consumption frequencies between the IP group and those with a regular pulse (hereafter referred to as the RP group) (*p* = 0.022). There was no significant difference in the degree of physical activity between the two groups.

### 3.2. Clinical Data Comparisons

None of the clinical characteristics or laboratory findings listed in Table 2 were statistically significantly different between the IP and RP groups, although the difference in the waist circumference approached the significance level with a *p* value of 0.065 (87.6 for the IP groups versus 84.9 for the RP group). The difference in the heart rate was associated with the second-lowest *p* value (66.0 for the IP group versus 69.2 for the RP group, *p* = 0.085), although it was not significant.

### 3.3. Pulmonary Function Test and Irregular Pulse

FVC/pFVC was significantly lower (85.9% versus 90.6%) (*p* = 0.005), and the prevalence of patients with FVC/pFVC < 80% was higher (42.7% versus 19.3%) (*p* < 0.001) in the IP group (Table 3). However, there were no differences in the mean FEV_1_/FVC values or the prevalence of FEV_1_/FVC <0.7 between the two groups. FEV_1_ was reduced (2.03 versus 2.14) (*p* = 0.040), and FEV_1_/pFEV_1_ was lower (88.7% versus 93.3%) (*p* = 0.027) in the IP group. Based on the interpretation of the PFT guidelines [16], subjects in the IP group more frequently showed restrictive (22.4% versus 12.4%) and obstructive patterns (37.5% versus 29.9%) and less frequently showed a normal pattern (40.1% versus 57.7%) compared to the RP group (*p* = 0.037).

### 3.4. The Effects of Reduced Pulmonary Function on IP

FVC/pFVC < 80% was associated with IP (aOR = 2.68, 95% CI: 1.50–4.80, *p* = 0.001) after adjustment for covariates (Table 4). The subjects with lower FEV_1_ were more likely to have an IP compared with those with higher FEV_1_. FVC/pFVC and FEV_1_/pFEV_1_ were also inversely associated with IP (FVC/pFVC: aOR = 0.976, 95% CI: 0.956–0.997, *p* = 0.023; FEV_1_/pFEV_1_: aOR = 0.986, 95% CI: 0.973–0.999, *p* = 0.040, respectively). Subjects with a restrictive or obstructive pattern had a significantly higher risk of IP than those with normal spirometry results (aOR = 1.95, 95% CI: 1.02–3.72, *p* = 0.043).

## 4. Discussion

Pulse palpation and optional ECG for those with an IP could help identify the associated arrhythmia, especially AF, which is the most beneficial diagnosis for the early prevention of stroke in asymptomatic and undiagnosed elderly subjects. Inpatient measurement of vital signs, such as pulse palpation, is usually performed by nurses or physician assistants. It would be important for those performing pulse palpation to be aware of the value of IP in detecting new AF and preventing stroke in an early and timely manner. The present study showed that the lowest quartile of FEV_1_, FVC/pFVC < 80%, and a restrictive or obstructive pattern based upon spirometry interpretation were significant risk factors of IP. Therefore, clinical nurses should pay particular attention to patients with more severely decreased pulmonary function or those already diagnosed with COPD or restrictive lung disease when performing pulse palpation. In contrast to inpatients who are carefully monitored by nurses, elderly community dwellers do not commonly have the opportunity to have their pulse checked by a professional on a daily or even weekly basis. Fortunately, it has been reported that a smartphone can be used to check for an IP at home without visiting a hospital [17,18]. Wearable devices such as a smartwatch are also now able to evaluate heart rhythms or irregular pulses with an improving accuracy [19]. This is particularly promising for elderly subjects because information technology applications will enable them to self-monitor for an IP at home without the aid of medical staff. Elderly people who are not accustomed to new technology, such as smartphones, could be assisted by family members. The appropriate application of such technology will enhance the timely detection of AF, even in elderly community members who are not hospitalized, especially those with reduced pulmonary function.

Reduced FEV1 and obstructive pulmonary disease are associated with a higher incidence of AF. The Atherosclerosis Risk in Communities (ARIC) study reported that impaired pulmonary function was correlated with higher AF incidence [20], and the Malmö Preventive Project found that impaired pulmonary function was an independent predictor of AF [21]. Therefore, people with impaired pulmonary function are part of an at-risk population that would benefit from more careful screening for AF. Alcohol consumption is also associated with AF. Recently, Kim et al. reported that frequent drinking per week is an important risk factor for AF using a Korean nationwide population-based study [22]. Our study showed similar findings—that an IP had significantly more participants with alcohol consumption more than 4 times per week. While the diagnosis of AF requires rhythm documentation using an ECG, the ECG is not a cost-effective, first-step screening tool. On the other hand, pulse palpation is reported to be a simple, noninvasive, first-step screening tool to guide ECG diagnostics for cardiac arrhythmias with a high sensitivity [5,23] and has been considered the evidence-based method of choice for screening for arrhythmia among individuals aged ≥65 by the European Society of Cardiology [7]. However, the American Heart Association/American College of Cardiology/Heart Rhythm Society (AHA/ACC/HRS) 2014 guidelines only mention pulse palpation as physical examination [1], and the AHA/ACC/HRS 2019 guidelines only focus on device detection [24]. For opportunistic screening, pulse palpation would be an especially useful method in older adults who had a risk factor for irregular pulse, such as poor pulmonary function. Putting this knowledge and our results together, we believe that elderly people with impaired pulmonary function would greatly benefit from attentive pulse palpation by healthcare providers as well as from self-palpation.

Undiagnosed AF is common, and opportunistic screening for silent AF is likely to be cost-effective in the elderly [9,25]. The screening of older populations yielded a prevalence of 2.3% for chronic forms of AF [26]. In the present study, the prevalence of IP was only 2.6%. This finding is consistent with the low prevalence of AF in the general population in Korea. In a healthy, asymptomatic rural Korean population, the prevalence of ECG-diagnosed AF was only 2.3% among 60- and 70-year-olds [27]. Since subjects with paroxysmal AF may be classified as having an RP, pulse palpation may only be able to screen those with persistent or permanent AF.

COPD is usually diagnosed using PFT; FEV1/FVC < 70% confirms the presence of persistent airflow limitation and thus of COPD [28]. The worldwide prevalence of COPD is estimated to be 3–11% [29]. However, the prevalence of history of COPD in the present study was just 1%. Therefore, the prevalence of COPD as assessed by PFT was higher than that determined from the patient responses on the questionnaires. This suggests that many subjects in this study might have undiagnosed COPD. Yoo et al. showed a similar trend, reporting that despite the high prevalence of COPD in Korea, the disease is underdiagnosed, and most COPD patients are under-treated [30].

### Limitations

This study has a few limitations. First, there were no available ECG data to confirm the presence or absence of arrhythmias and, if present, to verify the type of arrhythmia. Elderly subjects are susceptible to a wide range of different cardiac arrhythmias, many of which may be associated with radial pulse irregularities. Nonetheless, the aim of our study was to assess if there was any association between IP and reduced pulmonary function in the elderly but not to correlate IP with a real arrhythmia. Second, there were no data about the period of discontinuation of smoking in ex-smokers or the exact prevalence of cardiomyopathy or thyroid disease, which might have an influence on pulmonary function or the prevalence of arrhythmia, respectively. Lastly, although there was no significant difference in the percentage of participants taking antihypertensive medications between the two groups, there were no detailed data about specific types of antihypertensive drugs such as beta-blocker and calcium-channel blocker that could also have had some influence on pulse regularity. Despite the presence of some limitations, we believe that this study is significant, as no previous study has described the association between pulmonary function and IP identified by pulse palpation in the general population.

## 5. Conclusions

Our study showed that the elderly with reduced pulmonary function were more likely to have an irregular radial pulse than those with normal pulmonary function. This finding suggests that pulse palpation may be a good screening tool for cardiac arrhythmia in older adults with reduced pulmonary function. Healthcare providers, especially nurses and physician assistants, should pay close attention to assessing for the presence or absence of IPs and monitor for arrhythmias in older patients with reduced pulmonary function. Further studies will be necessary to determine if subjects <65 years of age with reduced pulmonary function also have an irregular radial pulse more often than those with normal pulmonary function.

## Figures and Tables

**Table 1:** General characteristics.

Characteristics	Irregular Pulse (*n* = 61)	Regular Pulse (*n* = 2286)	*p*
	*n* (%)	*n* (%)	
Age (yr)	73.0 (71.7–74.3)	72.2 (71.9–72.5)	0.214
Age ≥75 years	16 (32.2)	528 (31.0)	0.881
Sex (male)	29 (45.8)	979 (40.5)	0.493
Chronic disease			
Hypertension	45 (75.6)	1384 (62.6)	0.088
Diabetes mellitus	15 (28.1)	407 (18.9)	0.154
History of COPD	2 (2.0)	19 (1.0)	0.373
Congestive heart failure	2 (3.5)	47 (2.5)	0.660
Angina	2 (1.6)	112 (4.5)	0.126
Previous stroke	4 (6.2)	68 (3.1)	0.200
Previous myocardial infarction	2 (2.3)	50 (2.3)	0.987
Hyperlipidemia			
Hypercholesterolemia	8 (13.5)	502 (22.8)	0.150
Hypertriglyceridemia	5 (9.4)	331 (18.2)	0.151
Hypo-HDL cholesterolemia	15 (31.9)	614 (30.3)	0.855
Thyroid disease	0 (0.0)	112 (5.1)	0.155
Medication use, n (%)			
Antihypertensive drugs	37 (58.5)	1152 (51.2)	0.477
Antidiabetic drugs (including insulin)	11 (21.4)	366 (16.0)	0.379
Lipid-lowering drugs	4 (5.7)	327 (12.8)	0.136
Cigarette smoking status			0.703
Current smoker	8 (10.1)	254 (11.7)	
Ex-smoker	17 (32.3)	627 (26.8)	
Non-smoker	34 (57.6)	1367 (61.5)	
Alcohol consumption frequency			0.022
≤1 time/month	44 (74.6)	1590 (70.8)	
2–4 times/month	2 (3.4)	284 (12.7)	
2–3 times/week	3 (5.1)	184 (8.2)	
≥4 times/week	10 (16.9)	187 (8.3)	
Amount of alcohol			0.611
None	32 (57.9)	1051 (49.0)	
1–4 servings	20 (34.6)	977 (42.0)	
5–9 servings	6 (5.8)	185 (7.6)	
≥10 servings	1 (1.7)	34 (1.4)	
Physical activity			
Walking	21 (27.6)	885 (38.1)	0.202
Moderate activity	2 (6.6)	207 (9.1)	0.640
Strenuous activity	3 (4.0)	204 (8.2)	0.205

COPD: chronic obstructive pulmonary disease, HDL: high-density lipoprotein. Continuous variables are presented as the mean (95% confidence interval), and dichotomous variables are presented as n (%).

**Table 2:** Clinical data.

Characteristics	Irregular Pulse (*n* = 61)	Regular Pulse (*n* = 2286)	*p*
Body mass index, kg/m^2^	24.8 (23.9–25.8)	24.2 (24.0–24.4)	0.168
Waist circumference, cm	87.6 (84.7–90.5)	84.9 (84.2–85.5)	0.065
Blood pressure, mmHg			
Systolic	130.8 (125.8–135.9)	130.2 (129.3–131.1)	0.811
Diastolic	72.1 (68.5–75.8)	74.3 (73.8–74.9)	0.235
Heart rate, bpm	66.0 (62.4–69.6)	69.2 (68.6–69.8)	0.085
Pulse pressure, mmHg	58.7 (54.4–62.9)	55.9 (55.2–56.6)	0.193
White blood cell count, ×10^3^/mm^3^	6.3 (5.6–7.1)	6.1 (6.0–6.3)	0.616
Fasting glucose, mg/dL	104.7 (98.4–110.9)	102.7 (101.6–103.9)	0.546
eGFR, mL/min/1.73 m^2^	76.3 (72.7–79.9)	78.9 (77.7–80.1)	0.165
Total cholesterol, mg/dL	185.7 (174.4–197.0)	193.0 (190.9–195.1)	0.208
HDL-C, mg/dL	46.8 (43.5–50.1)	46.6 (46.0–47.2)	0.875
Triglycerides, mg/dL	125.6 (106.7–144.6)	141.0 (136.7–145.3)	0.117

eGFR: estimated glomerular filtration rate, HDL-C: high-density lipoprotein cholesterol. Continuous variables are presented as the mean (95% CI).

**Table 3:** Spirometry data.

Characteristics	Irregular Pulse (*n* = 61)	Regular Pulse (*n* = 2286)	*p*
FVC, L	2.84 (2.67–3.01)	2.95 (2.91–2.99)	0.208
Quartile 1 (1.25–2.439)	20 (36.5)	562 (27.6)	0.210
Quartile 2 (2.44–2.899)	12 (21.6)	586 (26.0)	
Quartile 3 (2.90–3.529)	21 (30.5)	559 (23.8)	
Quartile 4 (3.53–5.46)	8 (11.4)	579 (22.6)	
FVC/predicted FVC, %	85.9 (82.6–89.2)	90.6 (89.9–91.2)	0.005
FVC/predicted FVC, *n* (%)			<0.001
≥80%	36 (57.3)	1837 (80.7)	
<80%	25 (42.7)	449 (19.3)	
FEV_1_/FVC	0.72 (0.70–0.75)	0.73 (0.73–0.74)	0.656
FEV_1_/FVC, n (%)			0.293
≥0.7	42 (62.5)	1636 (70.1)	
<0.7	19 (37.5)	650 (29.9)	
FEV_1_, L	2.03 (1.93–2.13)	2.14 (2.11–2.17)	0.040
Quartile 1 (0.74–1.82)	17 (33.3)	563 (28.2)	0.122
Quartile 2 (1.83–2.14)	19 (35.2)	565 (26.1)	
Quartile 3 (2.15–2.53)	17 (23.0)	587 (23.9)	
Quartile 4 (2.54–3.99)	8 (8.5)	571 (21.8)	
FEV_1_/predicted FEV_1_, %	88.7 (84.8–92.7)	93.3 (92.4–94.2)	0.027
Interpretation, *n* (%)			0.037
Normal	27 (40.1)	1334 (57.7)	
Restrictive	15 (22.4)	302 (12.4)	
Obstructive	19 (37.5)	650 (29.9)	

FVC: forced vital capacity, FEV_1_: forced expiratory volume in 1 s. Continuous variables are presented as the mean (95% confidence interval), and dichotomous variables are presented as *n* (%).

**Table 4:** Univariate and multivariate logistic regression analysis of pulmonary function for irregular pulse.

Characteristics	Univariate Logistic Models			Multivariate Logistic Models		
	Unadjusted OR	95% CI	*p*	Adjusted OR ^†^	95% CI	*p*
FVC, L						
Quartile 1 (1.25–2.43)	2.62	1.04–6.57	0.041	2.57	0.96–6.88	0.061
Quartile 2 (2.44–2.89)	1.64	0.55–4.91	0.378	1.65	0.51–5.36	0.404
Quartile 3 (2.90–3.52)	2.53	0.94–6.82	0.066	2.41	0.89–6.51	0.082
Quartile 4 (3.53–5.46)	1			1		
FVC/predicted FVC, %	0.971	0.952–0.991	0.005	0.976	0.956–0.997	0.023
FVC/predicted FVC < 80%	3.11	1.73–5.61	<0.001	2.68	1.50–4.80	0.001
FEV1, L						
Quartile 1 (0.74–1.82)	3.04	1.15–8.06	0.025	3.09	1.02–9.37	0.047
Quartile 2 (1.83–2.14)	3.47	1.30–9.23	0.013	3.55	1.24–10.13	0.018
Quartile 3 (2.15–2.53)	2.47	0.97–6.30	0.059	2.44	0.90–6.59	0.079
Quartile 4 (2.54–3.99)	1			1		
FEV_1_/predicted FEV_1_, %	0.985	0.973–0.998	0.022	0.986	0.973–0.999	0.040
Interpretation						
Normal	1			1		
Restrictive	2.61	1.22–5.58	0.014	2.31	1.01–5.27	0.047
Obstructive	1.81	0.88–3.70	0.105	1.86	0.89–3.85	0.097
Restrictive or obstructive	2.04	1.09–3.83	0.026	1.95	1.02–3.72	0.043
History of COPD	2.14	0.47–9.64	0.468	2.56	0.57–11.60	0.221

COPD: chronic obstructive pulmonary disease, FVC: forced vital capacity, FEV1: forced expiratory volume in 1 s. ^†^ Odds ratios were obtained from a logistic regression model adjusted for age, sex, body mass index (BMI), waist circumference, chronic diseases, thyroid disease, medications, smoking status, alcohol consumption, and physical activity.
